# Septated gallbladder: a rare congenital anomaly and its clinical implications

**DOI:** 10.1093/jscr/rjag343

**Published:** 2026-06-19

**Authors:** Sudhanvan Iyer, Philong Nguyen, Joshua Wang, Suimin Qiu, Cristiana Rastellini, Luca Cicalese

**Affiliations:** John Sealy School of Medicine, The University of Texas Medical Branch, 301 University Boulevard, Galveston, TX 77555, United States; John Sealy School of Medicine, The University of Texas Medical Branch, 301 University Boulevard, Galveston, TX 77555, United States; John Sealy School of Medicine, The University of Texas Medical Branch, 301 University Boulevard, Galveston, TX 77555, United States; Department of Pathology, The University of Texas Medical Branch, 301 University Boulevard, Galveston, TX 77555, United States; Department of Neurobiology, The University of Texas Medical Branch, 301 University Boulevard, Galveston, TX 77555, United States; Department of Surgery, The University of Texas Medical Branch, 301 University Boulevard, Galveston, TX 77555, United States

**Keywords:** Septated gallbladder, atypical cholecystitis, congenital biliary anomaly, cholecystectomy, bile stasis, right upper quadrant pain

## Abstract

A septated gallbladder (SG) is a rare anatomical variant characterized by the presence of one or more fibrous or muscular septa dividing the lumen into multiple chambers. While an acquired form of septation has been discussed, most reported cases are treated as congenital variants. This study presents a case of SG identified during clinical evaluation and discusses its clinical significance. The patient is a 64-year-old woman with a history of right upper quadrant (RUQ) abdominal pain that radiates to the right scapula. She reports pain beginning 10 years ago as intermittent episodes but has progressed over the past two months. Upon review of prior imaging studies, a SG was identified, and the patient was scheduled for a laparoscopic cholecystectomy. The presence of septa was confirmed intraoperatively. Following the procedure, her symptoms resolved completely. Awareness of SG is essential for clinicians and radiologists to prevent misdiagnosis and guide appropriate management. While incidental, symptomatic cases benefit from surgical intervention.

## Introduction

A septated gallbladder (SG) is an uncommon anatomical variation in which fibrous or muscular septa partially or completely divide the gallbladder lumen into multiple chambers [1–4]. The prevalence of SG remains poorly defined due to its rarity and the limited number of reported cases. The literature indicates that SG is a rare congenital anomaly, with only 56 cases reported since 1963, with the last reported case being in June 2021 [1]. This condition can lead to bile stasis, gallstone formation, and chronic inflammation, which may mimic more common biliary pathologies such as cholelithiasis or cholecystitis [2]. Understanding the embryological development, clinical manifestations, and potential complications of a SG is essential for accurate diagnosis and appropriate management.

Embryologically, the gallbladder develops from the hepatic diverticulum near the fourth week of gestation [5, 6]. Any disruption in the vacuolization process can lead to incomplete lumen formation, resulting in congenital septa [5, 6]. While some cases remain asymptomatic, others may present with chronic pain and digestive issues due to impaired bile flow [2]. Given the nonspecific clinical presentation, proper recognition of this anomaly through imaging is crucial to prevent unnecessary interventions or delayed treatment. Furthermore, considering the potential impact of bile stasis, there is growing interest in assessing the long-term consequences and possible associations with other hepatobiliary disorders.

## Case presentation

### Patient history

Patient is a 64-year-old female with a medical history of right upper quadrant (RUQ) abdominal pain. She reports that the pain began around 10 years ago, initially intermittent, becoming daily for the past two months and associated with post prandial worsening, bloating, nausea, vomiting, and diarrhea. Over the past few months, the patient reports an unintentional 9 pounds weight loss.

### Findings

Laboratory exams revealed normal liver function tests (Total bilirubin, AST, ALT and Alkaline phosphatase) as well as Amylase and Lipase. A computerized tomography (CT) scan and an ultrasound (US) of the abdomen reported the absence of gallstones or intra and extra hepatic bile ducts dilatation but made no mention of other anomalies. However, an independent review of the imaging performed by the surgeon revealed a single linear septation traversing the gallbladder lumen in a transverse orientation, partially divided the gallbladder into two compartments (Figs 1 and 2). This septation appeared complete without wall thickening. While CT confirmed the absence of additional biliary tract anomalies, US provided the clearest delineation of the internal septation. Given the chronic and post-prandial nature of the patient’s symptoms, alternative etiologies including biliary dyskinesia, chronic peptic ulcer disease, and functional dyspepsia were considered. However, these diagnoses were considered less likely due to normal lab studies and the absence of additional structural abnormalities on imaging.

**Figure 1:**
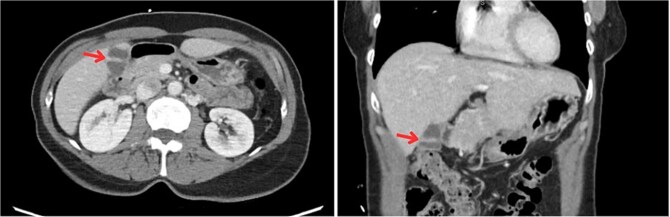
CT imaging of the abdomen showing a SG (arrows). Left: Axial view demonstrating an internal septation within the gallbladder. Right: Coronal view confirming the presence of a septation dividing the gallbladder lumen.

**Figure 2:**
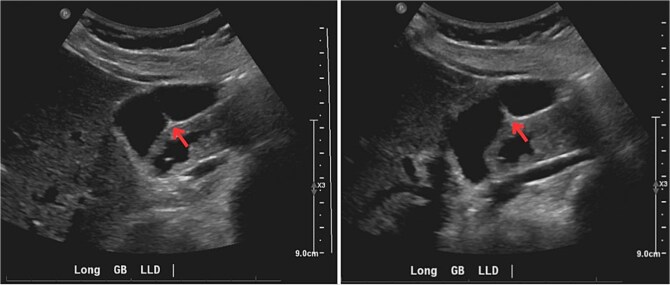
Longitudinal US images of the gallbladder demonstrating a septation (arrows). Left and right: Two separate views in the left lateral decubitus (LLD) position showing an internal echogenic band traversing the gallbladder lumen, consistent with a gallbladder septation.

### Management and outcome

The patient previously had a HIDA scan performed 5 years prior at an outside hospital showing a gallbladder with 98% ejection fraction. Given the patient’s symptoms and imaging findings, a repeat HIDA scan was offered, and a laparoscopic cholecystectomy was recommended, with the patient informed that symptom resolution could not be guaranteed. The patient refused a repeated HIDA scan and instead opted for laparoscopic cholecystectomy. The gallbladder was surgically removed laparoscopically without complication. Intraoperatively, the septated configuration did not obscure the field of view and the cystic duct and artery were identified without difficulty. Despite the unexpected contour of the gallbladder during dissection, there was no increase in operative difficulty and the presence of septa in the gallbladder lumen was confirmed by pathology (Fig. 3). Histopathologic evaluation revealed a tan-pink to green gallbladder (7.9 × 3.3 × 0.6 cm) with a smooth serosal surface, a roughened adventitial surface, and no evidence of chronic cholecystitis. The cystic duct was clipped at the margin and inked blue. No periductal lymph node was grossly palpated and a line is grossly observed alongside the entire length the specimen, at the serosal surface. The specimen shows a tan-pink and velvety mucosa, with a wall thickness of 0.4–0.5 cm, and no grossly observed bile and calculi. No discrete masses or polyps were grossly observed. Mild chronic inflammation was noted with a septum that is likely due to epithelial hyperplasia. At follow up, the patient reported complete resolution of the symptoms.

**Figure 3:**
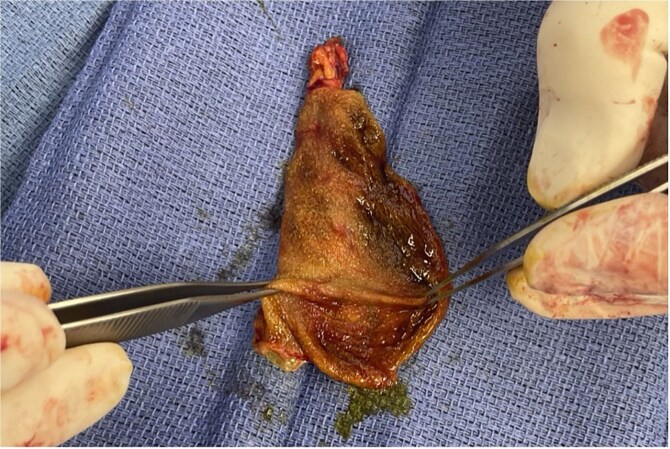
Image of the gallbladder specimen following cholecystectomy indicating a septation.

## Discussion

The gallbladder arises from the hepatic diverticulum and plays a crucial role in the digestive process by storing and concentrating bile [6, 7]. Disruptions in normal lumen formation during embryologic development may leave persistent intraluminal septa [6]. Additional theories have been proposed in the SG literature, including ‘wrinkling’ and ‘Phrygian cap’-related mechanisms in which rapid growth and external constraints contribute to folding and fusion of mucosal surfaces with subsequent septation formation [8]. Although acquired septation has been discussed (e.g. from chronic inflammation and fibrosis), the majority of reported septate cases are treated as congenital variants. Regardless of origin, SGs can predispose individuals to biliary stasis, recurrent colicky pain, and an increased likelihood of gallstone formation by promoting the concentration and precipitation of cholesterol and other substances in bile salts [2]. Despite existing theories regarding the pathophysiology of SG, the precise mechanism by which it produces symptoms is not fully understood. One proposed explanation is that internal septations may interfere with gallbladder contraction, leading to incomplete emptying and bile stasis within each chamber [9]. This stasis may then work to increase intraluminal pressure or result in intermittent obstruction which can cause recurrent pain. While symptoms resolved following cholecystectomy in our patient, this association remains inferential given the rarity of the anomaly and the absence of routinely performed HIDA scans in existing literature.

Reported presentations range from incidental discovery to chronic biliary-type symptoms. Across published cases, right upper quadrant or epigastric pain is the most common symptom, frequently accompanied by nausea/vomiting or postprandial worsening; however, atypical symptoms, such as IBS-like symptoms, have also been described to contribute to diagnostic delay [8–10]. Importantly, laboratory studies may be normal, and gallstones may be absent. These features can lead clinicians away from a biliary source despite persistent, meal-related right upper quadrant pain [2, 11]. Additionally, our patient had a past medical history significant for GERD, diverticulosis, osteopenia, postmenopausal atrophic vaginitis, and postmenopausal HRT. No malignancy was present following evaluation, and all conditions were followed and treated by other providers. Their postoperative clinic visits also demonstrated complete resolution of preoperative symptoms. Our patient’s decade-long symptom history with recent progression to daily postprandial right upper quadrant pain, scapular radiation, and associated gastrointestinal symptoms was ultimately recognized only after careful review of imaging.

US may reveal echogenic septa transversing the lumen, while CT and MRCP provide more detailed evaluation of septal extent and associated biliary anomalies [12, 13]. However, as in this case, SG may be missed when imaging reports focus solely on gallstones or ductal dilation, underscoring the importance of thorough image review.

Because SG is rare, there are no standardized management guidelines, and treatment is generally symptom-based. Observation is reasonable for asymptomatic patients; however, cholecystectomy is commonly recommended for persistent biliary-type symptoms [14, 15]. Reported outcomes following cholecystectomy are quite favorable as one review cited symptom relief in 17 of 25 cases after cholecystectomy [15]. Similar to previously reported symptomatic cases without cholelithiasis, our patient experienced complete symptom resolution following cholecystectomy. Compared with earlier reports, this case demonstrates the value of combining multimodal imaging with careful radiologic review when laboratory findings and initial studies are not diagnostic in the setting of persistent symptoms. This aligns with our patient’s outcome, supporting SG as a clinically meaningful etiology of chronic biliary symptoms even in the absence of stones.

## Conclusion

This case highlights two practical points. First, SG should remain on the differential for chronic, postprandial right upper quadrant pain despite normal laboratory studies and negative gallstone imaging. Second, careful radiologic review when symptoms are persistent and biliary in character can reduce delays in diagnosis and definitive management. Future work through multicenter imaging and pathology studies is needed to better define prevalence, characterize associated anomalies, and identify which symptomatic patients are most likely to benefit from surgery.
